# Modified T^2^ Statistics for Improved Detection of Aided Cortical Auditory Evoked Potentials in Hearing-Impaired Infants

**DOI:** 10.1177/23312165231154035

**Published:** 2023-02-27

**Authors:** Michael Alexander Chesnaye, Steven Lewis Bell, James Michael Harte, Lisbeth Birkelund Simonsen, Anisa Sadru Visram, Michael Anthony Stone, Kevin James Munro, David Martin Simpson

**Affiliations:** 1 59622 Institute of Sound and Vibration Research, Faculty of Engineering and the Environment, University of Southampton, Southampton, UK; 2 Interacoustics Research Unit, Technical University of Denmark, Lyngby, Denmark; 3 Eriksholm Research Centre, Snekkersten, Denmark; 4 Manchester Centre for Audiology and Deafness, School of Health Sciences, 5292 University of Manchester, Manchester, UK; 5 Manchester University Hospitals NHS Foundation Trust, Manchester Academic Health Science Centre, Manchester, UK

**Keywords:** evoked potentials, aided CAEP detection, hearing-impaired infants, Hotelling's T^2^ test

## Abstract

The cortical auditory evoked potential (CAEP) is a change in neural activity in response to sound, and is of interest for audiological assessment of infants, especially those who use hearing aids. Within this population, CAEP waveforms are known to vary substantially across individuals, which makes detecting the CAEP through visual inspection a challenging task. It also means that some of the best automated CAEP detection methods used in adults are probably not suitable for this population. This study therefore evaluates and optimizes the performance of new and existing methods for aided (i.e., the stimuli are presented through subjects’ hearing aid(s)) CAEP detection in infants with hearing loss. Methods include the conventional Hotellings T^2^ test, various modified q-sample statistics, and two novel variants of T^2^ statistics, which were designed to exploit the correlation structure underlying the data. Various additional methods from the literature were also evaluated, including the previously best-performing methods for adult CAEP detection. Data for the assessment consisted of aided CAEPs recorded from 59 infant hearing aid users with mild to profound bilateral hearing loss, and simulated signals. The highest test sensitivities were observed for the modified T^2^ statistics, followed by the modified q-sample statistics, and lastly by the conventional Hotelling's T^2^ test, which showed low detection rates for ensemble sizes <80 epochs. The high test sensitivities at small ensemble sizes observed for the modified T^2^ and q-sample statistics are especially relevant for infant testing, as the time available for data collection tends to be limited in this population.

## Introduction

Approximately 1 in 1000 infants are born each year with permanent bilateral hearing loss (Davis et al., 1997; Fortnum et al., 1997). When left untreated, hearing loss has been associated with a cascade of long-lasting detrimental effects (Hogan et al., 2014; Idstad et al., 2019; Moeller 2007; Ramkalawan & Davis, 1992; Stevenson et al., 2015; Theunissen et al., 2014; Yoshinaga-Itano et al. 1998). It is therefore important that hearing loss is diagnosed and treated (e.g., by fitting a hearing aid) at a young age, ideally within the first 3 months of life (Ching et al., 2013; Yoshinaga-Itano et al., 1998). However, this poses some challenges, as behavioral hearing tests cannot be reliably performed until a developmental age of 7–9 months (Widen, 1993). Early audiological intervention procedures have therefore been built around objective measures of hearing that do not require voluntary responses, such as auditory evoked potentials (AEPs).

AEPs are defined as changes in brain activity in response to acoustic stimuli (Picton, 2011). They are typically recorded non-invasively using the electroencephalogram (EEG), and comprise a series of peak and trough voltage amplitudes falling within the first ∼1 s interval following stimulus onset. The AEP can also be sub-categorized into various components based on the underlying neural generators along the auditory pathway. These components can be identified by the latencies (time following stimulus onset) of various peaks and troughs; thus the cochlear microphonic, the auditory brainstem response (ABR), the mid-latency response, and the cortical auditory evoked potential (CAEP) have been defined (e.g., Picton 2011), in order of their peak and trough latencies.

For evaluating hearing function in infants, the most commonly used AEPs are the ABR and the CAEP (Lightfoot et al., 2016; Lightfoot et al., 2019). A potential advantage for the ABR over the CAEP is that it can be reliably recorded during sleep (e.g., Jewett & Williston, 1971) and is unaffected by attention (Picton & Hillyard, 1974). The drawback is that it cannot be reliably recorded in some patients, such as those with auditory neuropathy spectrum disorder (Roush et al., 2011). Furthermore, when testing patients with their hearing aids on, results may not reflect patients’ hearing, as the hearing aids do not respond well to the rapid presentation rate of ABRs which confuse noise reduction algorithms. It might also be argued that the ABR provides a limited assessment of the integrity of the auditory pathway, as it represents neural activity generated by just the early components of the pathway. This contrasts with the CAEP which represents neural activity generated by thalmo-cortical brain regions.

Early audiological assessment using CAEPs is a challenging procedure, both in terms of evoking the CAEP, and in accurately detecting it within clinically acceptable test times. In terms of signal-to-noise ratios (SNRs), the CAEP has typical peak amplitudes in the 5–10 µV range (e.g., Munro et al., 2020; Picton 2011), whereas the background activity tends to be in the ∼50 µV range after pre-processing and artefact rejection. It is therefore common practice to record an ensemble of CAEPs following repeated stimuli, and to average the recorded waveforms to reduce “noise.” Clinicians are then given the task to visually inspect the waveforms, and to determine whether a CAEP is absent or present. This is especially challenging for hearing-impaired infants, as waveforms can vary substantially across individuals due to different degrees of hearing-impairment (Oates et al., 2002) and/or lack of maturation of the auditory system (Ponton & Eggermont, 2007). As such, it is not always clear to the examiners what to look for in the displayed waveforms. Various objective methods have therefore been proposed to assist the clinicians with this task, and improve the accuracy and efficiency of the test. However, the challenges for visual CAEP inspection arising from the variability in infant responses also impact objective detection methods: infant responses can be considerably different from adult responses, and template matching methods that performed best in adults (Chesnaye et al., 2021a) are unlikely to work well in this young population.

Perhaps the most commonly used objective method for CAEP detection is the Hotelling's T^2^ (HT^2^) test, applied in the time domain (Golding et al., 2009). The HT^2^ test was previously shown to have a test sensitivity similar to experienced clinicians when detecting CAEPs in infants (Carter et al., 2010), and has some desirable properties in terms of statistical power (Anderson, 2003). A drawback for the HT^2^ test, however, is that it is known to suffer from low test sensitivities at relatively small sample sizes. The latter is also known as the “large p small n” problem (Li et al., 2017) or the “effect of high dimension,” and has been hypothesized to be due to poor estimates of the feature covariance matrix (Bai & Saranadasa, 1996), which is a key component in calculating the T^2^ statistic. The low test sensitivity for the HT^2^ test was previously also observed for CAEP detection in adults with normal hearing where it was outperformed by various alternative detection methods for ensemble sizes of ∼40 epochs or less (Chesnaye et al., 2021a). Note that high test sensitivities for small ensemble sizes are of particular interest for infant CAEP detection as the time windows for high quality data collection tend to be limited.

The present study aims to overcome the low test sensitivity of the HT^2^ test by exploiting the correlation structure underlying the data to allow improved feature covariance matrix estimation (section “Modified T2 statistics”). The T^2^ statistics with modified covariance matrices were compared against the conventional HT^2^ test, along with various alternative detection methods from the literature. To keep this work concise, results are presented for just the best-performing methods from the literature, which were the modified q-sample uniform scores statistics from Stűrzebecher et al. (1999) and Cebulla et al. (2006). Methods that were excluded from the results section are instead considered in the Discussion. This includes the previously best-performing adult CAEP detection method, that is, a template-based dynamic time warping (DTW) approach (Chesnaye et al., 2021a) and some of its variations. Comparisons were also drawn with visual inspection results from three experienced audiologists. Finally, test significance was evaluated using a recently developed frequency-domain bootstrap approach (Chesnaye et al., 2021b), and the assessment was carried out using aided CAEP measurements from 59 infant hearing aid users, as well as simulated signals.

## Methods

This section describes the infant CAEP data and the objective detection methods, after which the procedures for evaluating test performance are described, which include an assessment of specificity, sensitivity, and reliability.

### CAEP Data

Aided CAEP measurements were previously recorded from 103 infants (aged 3–7 months) with mild to profound bilateral hearing loss. Standard otoscopy and tympanometry examinations were carried out, and hearing aids were checked to confirm that these were working as intended. For the CAEP examination, the infants were seated in a soundproof booth on their caregiver's lap, approximately 1.1 m directly in front of an Eminence Alpha-6A 8 Ω loudspeaker (Eminence Speaker LLC, Eminence, KY). CAEPs were then evoked using 70 ms duration synthetic speech tokens with 10 ms duration raised-cosine onset and offset ramps, presented at a rate of approximately 0.9 Hz. These synthetic stimuli comprised either harmonics 6–11 of a tone with a 140-Hz fundamental—henceforth denoted as the mid-frequency stimulus, or MF—or an inharmonic series of closely-spaced tones spanning 2800–4500 Hz, to produce a fricative-like phoneme—henceforth denoted as the mid-high frequency stimulus, or HF. The levels of the individual components in each stimulus were chosen so as to produce a uniform excitation of the cochlea in a bandwidth that was at least the width of four normal auditory filters (Glasberg & Moore, 1990). The relative levels of the stimuli were referenced to the power contained within the same bandwidth of the International Speech Test Signal (ISTS, Holube et al., 2010) compared to the overall level of the full-bandwidth level of the ISTS. The relative levels were −14.5 and −20.6 dB for the MF and HF signals, respectively, compared to the full-bandwidth level. For clarity, we reference the presentation levels of each stimulus to the full bandwidth level of the ISTS from which they would have been derived, and label this the “Speech Reference Level” (SpRefL). So, for the MF stimulus presented at 65 dB SpRefL, the actual level of the stimulus during its steady-state portion was (65–14.5)  =  50.5 dB SPL. A more extensive description and rationale for the design of the stimuli is described in Stone et al. (2019).

The stimuli were presented at 65 dB SpRefL (for 66 infants), 75 dB SpRefL (29 infants), and either 79 dB SpRefL (MF stimulus, 8 infants) or 85 dB SpRefL (HF stimulus, 8 infants). Throughout the CAEP test, an experienced paediatric audiologist aimed to keep the infant's attention facing towards the loudspeaker by using a selection of silent toys. The stimuli were then presented repeatedly until 20 artefact-free epochs had been recorded using an artefact rejection threshold of  ± 110 μV and a band-pass filter of 1–100 Hz. A total of four blocks for the MF stimulus and four for the HF stimulus were presented using an interleaved approach. This procedure was then repeated, with the stimulus order reversed, giving 160 artefact-free epochs, per stimulus (although in some recordings there were 159 epochs). The full CAEP test procedure was repeated within seven days. If the repeat session was on the same day, a break of at least 1 h was given before starting the next session. Finally, CAEP measurements were made using electrodes placed at the high forehead (Fpz; active electrode), the right mastoid (reference electrode), and the left mastoid (ground electrode), and were recorded at a sampling rate of 30 kHz (downsampled to 500 Hz for further analysis) using the Eclipse system (Interacoustics, Middelfart, Denmark). Data were filtered offline from 1 to 15 Hz using 3rd-order Butterworth filters. Ethics approval was obtained from the North West National Research Ethics Service Ethics Committee (reference 15/NW/0736).

#### Visual Reinforcement Audiometry

To determine the audibility of the stimuli presented during the CAEP sessions, aided behavioral hearing thresholds to the same stimuli were estimated using visual reinforcement audiometry (VRA; BSA, 2014). As VRA requires behavioral conditioning, it was performed several months after the CAEP sessions. Infants were aged between 7.4 and 21 months, with a mean age of 10.8 months. VRA was performed by two experienced pediatric audiologists who followed the British Society of Audiology recommended procedure as a guideline (BSA, 2014). During the VRA sessions, stimuli were presented at a rate of 4 Hz using the same set-up previously described for the aided CAEP sessions. Infants were first conditioned at a high test level with simultaneous presentation of a visual reinforcer. An adaptive procedure was then used to find threshold in 5 dB step sizes. One audiologist kept the child's quiet attention in the room, and the other controlled the sounds from the observation room, and judged when a response was present. Intermittent control trials and long gaps were included to judge for false responses, but the tester was not blind to these. At the VRA session, standard hearing aid checks, otoscopy examinations, and tympanometry examinations were again carried out. If hearing aid settings had changed since the CAEP sessions, the VRA was performed while wearing temporary hearing aids of the same model as used during the CAEP session and set to their previous settings. Hearing aid settings were also checked in a test box for consistency between the CAEP and VRA sessions. The decibel Sensation Level (dB SL) was estimated as CAEP presentation level minus the aided VRA-estimated behavioral threshold, both recorded in dB SpRefL in the sound field.

#### Which Participants to Include

To accurately evaluate the performance of the detection methods, it is important that the estimated audibility of the CAEP stimuli is accurate. Participants were therefore excluded if the dB SL of the CAEP stimulus could not be accurately estimated. More specifically, the criteria for exclusion were: (i) middle ear dysfunction during CAEP or VRA test sessions, which could affect the recorded sensation level, (ii) progressive or fluctuating hearing loss between the sessions, and/or (iii) unreliable or missing behavioral thresholds. Additionally, one infant was excluded who was not aided at the time of the CAEP test, and another who used a bone conduction hearing aid. This resulted in a total of 59 and 57 infants with reliable dB SL estimates for the MF and HF stimuli, respectively (Table 1). Two recordings were available per participant giving a total of 118 recordings from 59 participants for MF, and 114 recordings from 57 infants for HF.

**Table 1.** An overview of the number of recordings available for the analysis, per dB SL category and per stimulus type.

Stimulus	Decibel sensation level (dB SL)			
	<0	0–10	10.1–20	>20
MF	22	42	36	18
HF	22	34	44	14

### Objective detection methods

**Figure 1.**
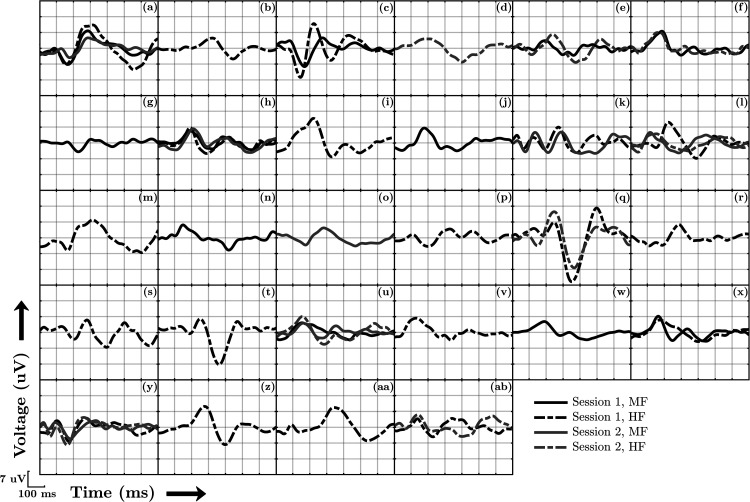
All 45 CAEP template waveforms where a CAEP was deemed present by all three audiologists. Each panel shows waveforms from a single infant, and includes waveforms evoked by either the mid frequency (MF) or the mid-high frequency (HF) stimulus from sessions one or two, giving a maximum of four waveforms per infant. These waveforms were used to emulate CAEPs in simulations for the sensitivity assessment. Note that these waveforms were obtained by averaging across ensembles of 160 epochs, whereas the audiologists were originally reviewing coherent average replicates of 80 epochs.

All objective detection methods were applied to the 700 ms windows following stimulus onset, as some infants had relatively late and/or long-lasting responses (see also Figure 1). For the frequency-domain detection methods, all 700 ms windows following stimulus onset were transformed to the frequency domain using the fast Fourier transform (FFT), giving a spectral resolution of ∼1.43 Hz. Note that the FFT was applied to the individual epochs, and not to their coherent average data.

#### The one-sample Hotelling's T^2^ test

The one-sample Hotelling's T^2^ (HT^2^) test is the multivariate equivalent to Student's one-sample t-test (Hotelling, 1931), and is one of the more commonly used methods for CAEP detection (e.g., Carter et al., 2010; Chang et al., 2012; Chesnaye et al., 2021a; Golding et al., 2009; Van Dun et al., 2012). When applied in the time domain, features consist of mean voltages, taken across short time intervals within epochs, henceforth referred to as “voltage means.” Extracting *Q* voltage means from an ensemble of *N* epochs gives an *N*x*Q*-dimensional matrix of features, say **V**. A vector of *Q* mean feature values, say $\bar{x}$, is then found by averaging down the *Q* columns of **V.** The one sample HT^2^ test evaluates the hypothesis that the $\bar{x}$ values are equal to *Q* hypothesized values, say μ. Under the null hypothesis H0 of “no CAEP present,” the μ hypothesized values are all zero, because the mean of the recordings are shifted to zero due to the high-pass filter. The T^2^ statistic itself is given by (Rencher 2001):

Eq. 1 $$T^{2}=N(\bar{x}-\mu )S^{-1}(\bar{x}-\mu {)}^{H}$$

where $S^{-1}$ is the inverse of the covariance matrix of **V**, and ^H^ denotes Hermitian transpose. With respect to the choice for *Q*, additional simulations presented in the Supplemental Digital Content demonstrate a good performance when taking voltage means across 50 ms intervals, giving *Q*  =  14 for the 700 ms analysis window. The HT^2^ test applied to *Q*  =  14 voltage means is henceforth referred to as “T^2^_Time_.”

##### Frequency Domain Features for T^2^_Freq_

When the HT^2^ test is applied in the frequency domain—henceforth referred to as “T^2^_Freq_”—features are the real and imaginary parts of the Fourier components of *W* spectral bands (e.g., Chesnaye et al., 2018), and form the *N*x2*W*-dimensional feature matrix **V**. The mean feature vector $\bar{x}$ is then again found by averaging down the *2W* columns of **V**. Under the null hypothesis of “no CAEP present,” it is assumed that the phases of each spectral band are uniformly distributed between 0 and 2π, and hence that the real and imaginary parts are randomly distributed around zero. The hypothesized values to test against (i.e., μ) are therefore given by a 2*W*-dimensional vector of zeros. Additional simulations, presented in the Supplemental Digital Content, were carried out to determine which spectral bands to include in the analysis. Results show a good performance when using the 5.7, 4.3, 7.1, 2.9, 1.4, and 8.6 Hz bands (in rank order), which were the six top-ranking spectral bands in terms of their estimated SNRs.

##### Evaluating Test Significance

Statistical inference on the T^2^ statistic was carried out using conventional F-distributions, which can be derived from theory based on the assumption that epochs are independent, and that data are stationary and normally distributed. The T^2^ statistic is transformed into an F-statistic using $\frac{N-Q}{Q(N-1)}T^{2}$, which is F-distributed with v_1_ and v_2_ degrees of freedom under H0. When using *Q* voltage means, v_1_  =  *Q* and v_2_  =  *N*−*Q*, whereas when using *W* spectral bands, v_1_  =  2*W* and v_2_  =  *N*−2*W*. For the remaining detection methods, test significance was evaluated using a bootstrap, described below.

#### Modified T^2^ statistics

The aim for the modified T^2^ statistics is to prevent low HT^2^ test sensitivities at small sample sizes by exploiting prior knowledge of the correlation structure underlying the data, which allows an improved estimate of the feature covariance matrix. In the time domain, data is assumed to follow a Toeplitz covariance structure, whereas in the frequency domain, independence between spectral bands is assumed.

##### Time Domain Modification T^2^_Toep_

A time series with Toeplitz covariance implies that the expected covariance between any two data points is dependent only on their time difference (Mukherjee & Maiti, 1988), which is satisfied for random stationary signals. When Toeplitz covariance is met, then the covariance matrix of the voltage means (previously denoted by **S**) can be found from the autocorrelation function of the continuous recording (further described below). This contrasts with the usual method, where each value in **S** is estimated individually from pairs of columns in the epoched data, and thus from a much smaller set of samples. 

In what follows, note the distinction between voltage means extracted from the epochs, and the voltage means extracted from the continuous recording. For the voltage means extracted from the continuous recording, Toeplitz covariance implies the following property:

Eq. 2 $$E[\mathrm{cov}(\mathrm{xi},\mathrm{xi}+\lambda_{1})]=E[\mathrm{cov}(\mathrm{xi}+\lambda_{2},\mathrm{xi}+\lambda_{1}+\lambda_{2})]$$

where xi is the i^th^ voltage mean, cov denotes covariance, *E* is the expectation operator, and $\lambda_{1}$ and $\lambda_{2}$ denote time lags, which can take any integer value (in samples for digital signals). Note that the expected covariance is not dependent on time lag $\lambda_{2}$.

When Toeplitz covariance (Equation 2) is met, then covariance matrix **S** (i.e., the covariance matrix estimated from **V**) has the property that the diagonals (descending from left to right) are constant, and can be derived from the autocovariance function of the voltage means of the continuous recording. More specifically, the first row of **S** is given by the first *Q*−1 time lags of the autocovariance function using (Xiao & Wu, 2012):

Eq. 3 $$\gamma (\lambda_{1})=\mathrm{cov}(\mathrm{xi},\mathrm{xi}+\lambda_{1})$$

where time lag $\lambda_{1}$ takes values of $\lambda_{1}=0,1,2,\ldots ,Q-1$. The remaining rows of **S** are then rotated versions of the first row, which becomes evident when inspecting the following characteristic Toeplitz covariance structure (Mukherjee & Maiti, 1988):

Eq. 4 $$S_{Toep}=\left[\begin{array}{ccccc} \gamma (0) & \gamma (1) & \gamma (2) & \cdots & \gamma (Q-1)\\ \gamma (1) & \gamma (0) & \gamma (1) & \ddots & \vdots \\ \gamma (2) & \gamma (1) & \ddots & \ddots & \gamma (2)\\ \vdots & \ddots & \ddots & \gamma (1) & \gamma (1)\\ \gamma (Q-1) & \cdots & \gamma (2) & \gamma (1) & \gamma (0) \end{array}\right]$$

Note that the number of unknown parameters in $S_{Toep}$ is now *Q*, as opposed to *Q*(*Q*  +  1)/2 in **S**. The final test statistic, say T^2^_Toep_, is given by Equation 1, after replacing **S** with $S_{Toep}$:

Eq. 5 $$T_{\mathrm{Toep}}^{2}=(\bar{x}-\mu ){S_{Toep}}^{-1}(\bar{x}-\mu {)}^{H}$$

The degrees of freedom for this modified version of HT^2^ test differ from the conventional HT^2^ test, which prevents the convenient use of the F-statistic usually employed with HT^2^, but the bootstrap approach provides a simple, albeit computationally intensive, alternative (section “Frequency Domain Bootstrap”).

##### Frequency Domain Modification T^2^_Diag_

For the frequency domain HT^2^ modification, the asymptotic independence between spectral bands and real and imaginary components, expected from theory for stationary signals, is exploited. This leads to all covariances being zero, giving the following diagonal covariance matrix:

\rm \itEq. 6 $$S_{Diag}=\left[\begin{array}{ccccc} S1 & 0 & 0 & \cdots & 0\\ 0 & S2 & 0 & \ddots & \vdots \\ 0 & 0 & \ddots & \ddots & 0\\ \vdots & \ddots & \ddots & S2W-1 & 0\\ 0 & \cdots & 0 & 0 & S2W \end{array}\right]$$

where Si is the variance of the i^th^ feature, for example, S1 and S2 would be the variance of the real and imaginary parts, respectively, of the Fourier components of the 1st spectral band (the ∼1.43 Hz band), S3 and S4 would be the variances of the real and imaginary parts, respectively, of the second spectral band (∼2.66 Hz), etc.

Additional analysis presented in the Supplemental Digital Content shows that the SNR was not equal across spectral bands, which suggests that it may be beneficial to include band-specific weights, with larger weights being placed on the high SNR bands relative to the low SNR bands. The weighted T^2^ statistic is then given by:

\rm \itEq. 7 $$T_{\mathrm{Diag}}^{2}=(w\bar{x}-\mu )S_{Diag}^{-1}(w\bar{x}-\mu {)}^{H}$$

where **w** is a 2*W*-dimensional vector of weights. The weight vector was previously optimized in terms of statistical power in the Supplemental Digital Content (Table A.1), and was later simplified to the following rules of thumb: (i) All bands between 1 and 8 Hz received a weight of 1, (ii) all bands between 8 and 9 Hz received a weight of 0.75, and (iii) all bands between 9 and 15 Hz a weight of 0.5. As was the case with T^2^_Toep,_, the statistical significance of T^2^_Diag_ was evaluated using a bootstrap approach (section “Frequency Domain Bootstrap”).

#### Modified q-sample uniform scores test

The original q-sample uniform scores test (Mardia, 1972) can be used to test whether the phases of multiple spectral bands share the same underlying distribution. The modifications proposed by Sturzebecher et al. (1999) and Cebulla et al. (2006) consider the amplitudes of the spectral bands, in addition to the phases. The modifications are furthermore applied either to the actual values, or to the ranks of the phases and amplitudes, where the ranking is performed across all spectral bands and all epochs, that is, rank values (for either the phases or the amplitudes) range from 1 to *N*·*W*, where *W* is the number of frequency bands included in the analysis. The current study includes four variations: (i) QMod V1, applied to phase ranks and amplitude values, (ii) QMod V2, applied to phase ranks and amplitude ranks, (iii) QMod V3, applied to phase values and amplitude ranks, and (iv) QMod V4, applied to phase values and amplitude values. The test statistic, say QMod, is given by (Cebulla et al., 2006):

Eq. 8 $$\mathrm{QMod}=\overset{W}{\underset{\;j=1}{\sum }}\;[\;{\left(\overset{N}{\underset{i=1}{\sum }}\;r_{ij}\cos\frac{a_{ij}2\pi }{N\cdot W}\right)}^{2}+\;{\left(\overset{N}{\underset{i=1}{\sum }}\;r_{ij}\sin\frac{a_{ij}2\pi }{N\cdot W}\;\right)}^{2}\;]$$

Where $\;r_{ij}$ is either the rank or the actual value of the amplitude of the *j*^th^ spectral band from the *i*th epoch, and where $a_{ij}$ is either the rank or the actual value of the phase of the *j*^th^ spectral band from the *i*^th^ epoch. The modified q-sample statistics were applied to the 1.4, 2.8, 4.3, 5.7, 7.1, 8.6, and 10 Hz frequency bands, chosen based on additional simulations presented in the Supplemental Digital Content. The significance of QMod was evaluated using a bootstrap, as described next.

#### Frequency Domain Bootstrap

The frequency domain bootstrap (FDB) is an approach for generating many additional recordings, or “surrogates” (Chesnaye et al., 2021b; Dahlhaus & Janas, 1996). By applying the adopted detection method to a large number of surrogate recordings that satisfy the null hypothesis (i.e., no response present), a distribution of test statistics can be generated, henceforth the “bootstrapped distribution.” It is assumed that the bootstrapped distribution approximates the true null distribution underlying the test statistic. In order to obtain a good approximation, it is important that the surrogate recordings are representative of data under the null hypothesis, which requires certain data characteristics to be emulated, such as power and serial correlation between samples in each recording. In particular, for a given recording, say Xt, the FDB aims to generate surrogate recordings with similar power and serial correlation as Xt using the following steps (Chesnaye et al., 2021b):

1. Estimate the power spectral density (PSD) underlying Xt, say Pj, where *j* is the index of the frequency bin (*j*  =  1, 2, …, T). Welch's method (Welch, 1967) was used in the present study to estimate Pj (further details below).
2. Generate random surrogate PSDs by adding random variation to the estimated PSD through: $Pj^{*}=\hat{P}j\cdot \varepsilon j^{*}$, where $\hat{P}j$ is an estimate of Pj, and $\varepsilon j^{*}$ is randomly sampled from a standard exponential distribution with a mean of one (Dahlhaus & Janas, 1996). To clarify, note that the standard exponential distribution is related to the $\chi^{2}$ distribution through $0.5\chi_{2}^{2}=Exp(1)$ (Leemis & McQuestion, 2008), and that the $\chi^{2}$ distribution describes the sum of the squared real and imaginary parts (i.e., a sum of squares or the square of the amplitude) of the FFT components at frequency *j*.
3. Transform the random surrogate PSDs to magnitudes, and assign a random phase to each frequency bin where the phases are randomly sampled from a uniform distribution on the [−π, π] interval. For the time domain detection methods, take the inverse FFT to obtain time-domain surrogates.
4. Analyze the random surrogates with the detection method to generate a distribution of test statistics, which can be assumed to approximate the recording-dependent null distribution underlying the test statistic.

A crucial parameter underlying the performance of the FDB is the length of the sliding window within Welch's method, applied to Xt, which determines the spectral resolution and hence the “smoothness” of $\hat{P}j$. Smoothing helps to reduce variance in the $\hat{P}j$ estimate, and helps to mitigate stimulus-induced peaks, which would otherwise bias $\hat{P}j$ towards the PSD of the alternative hypothesis of “CAEP present,” as opposed to the null hypothesis of “no CAEP present.” The current study used a window length of 2 s, which was chosen based on pilot simulations (details not presented). Each bootstrapped distribution was generated from a total of 1000 surrogates. A more formal description along with an illustrative example can be found in Chesnaye et al. (2021b).

### Visual Inspection by Audiologists

The infant CAEP data were first evaluated by three experienced audiologists who determined whether a CAEP was present or absent in accordance with guidelines provided by the British Society of Audiology (Lightfoot et al., 2016). Data were presented to the audiologists as two replicates of the coherently averaged epoch where each replicate was obtained by averaging 80 epochs. The coherent average replicates were presented from −150 to  + 750 ms relative to stimulus onset, with the *y*-axis fixed at −15 to  + 15 μV. The examiners then made the decision of either (i) response present, (ii) response absent, or (iii) ambiguous. The ambiguous option was included to more accurately represent real-world test conditions where clinician's similarly have the choice to remain undecided, and potentially collect more data to resolve any ambiguities (Lightfoot et al., 2016). Data were furthermore presented to the examiners randomly, with no knowledge of subject ID or stimulus level, and with no knowledge of the output of the statistical detection methods. The aim for the visual inspection was to obtain a score to compare the performance of the objective detection methods against. Audiologists’ assessments also formed the basis for constructing a sample of CAEP waveforms, which were later used to emulate CAEP signals in simulations, as described in section “Test Sensitivity”.

### Test Specificity

The specificity of the detection methods was evaluated using false-positive rates (FPRs), defined as the rate at which H0 is rejected when H0 is true. Ideally, the FPR should equal the significance level of the test, also known as the nominal α-level, set here to 0.01. Data for the specificity assessment consisted of the inaudible (<0 dB SL) CAEP recordings, as well as simulated data to provide a more powerful assessment using a large number of tests where the ground truth of response absence was known.

#### Simulations

Data for the simulations consisted of stationary, Gaussian-distributed colored noise with spectral content similar to the inaudible (<0 dB SL) infant CAEP recordings. The colored noise was generated by filtering Gaussian white noise with all-pole filters, where the poles of the filters were given by the parameters of 20th-order autoregressive (AR) models. The AR parameters were estimated using the Yule-Walker approach (Hayes, 1996) with a new model being fitted to the EEG signals of each inaudible CAEP recording (44 in total). The resulting colored noise was then processed in a manner similar to the recorded EEG signals, that is, band-pass filtered from 1 to 15 Hz using a 3rd-order Butterworth filter, and structured into ensembles of either *N*  =  20, *N*  =  40, *N*  =  80, or *N*  =  160 approximately 1111 ms long epochs, corresponding to an approximately 0.9 Hz stimulus rate. The initial 700 ms windows of the ensembles were then analyzed with the detection methods. A total of 10,000 ensembles were simulated, per ensemble size.

#### Infant CAEP Data

The inaudible CAEP recordings were structured into ensembles of *N* epochs, where *N* again took values of either 20, 40, 80, or 160 epochs, to probe specificity with clinically more desirable small numbers of epochs. As the stimuli were deemed inaudible (<0 dB SL), no distinction was made between data from the MF and HF stimuli, nor between test sessions. There were sufficient data to assemble 347, 171, 83, and 44 ensembles, for *N*  =  20, 40, 80, and 160 epochs, respectively. The 700 ms post-stimulus windows of the ensembles were analyzed with the objective detection methods. For the visual assessments, FPRs were evaluated for the *N*  =  160 test condition, that is, 44 waveforms were visually inspected by each examiner.

#### Post-hoc Analysis

To determine whether the FPRs deviated significantly from the 0.01 α-level, 99% confidence intervals were constructed using binomial distributions. A binomial distribution represents the distribution of *m* “successful” Bernoulli trials out of *M* total trials performed. For the current analysis, a successful Bernoulli trial is defined as a false-positive, and hence has a theoretical probability equal to the nominal α-level of 1%. The total number of Bernoulli trials *M* is furthermore given by the total number of tests performed, and equals 10,000 for the simulations, which resulted in 99% confidence intervals of [0.0076, 0.0127] for α = 0.01. For the infant CAEP recordings, there were sufficient data for 44 tests (*N*  =  160), 83 tests (*N*  =  80), 171 tests (*N*  =  40), and 347 tests (*N*  =  20), giving 99% confidence intervals of [∼0, 0.081], [∼0, 0.055], [∼0, 0.038], and [∼0, 0.028], respectively.

### Test Sensitivity

Test sensitivity was evaluated using the true-positive rate, defined as the rate at which H0 is rejected when H0 is indeed false. Test sensitivity should ideally be as high as possible, albeit for a fixed FPR and ensemble size *N*. Test sensitivity was evaluated using the audible (≥0 dB SL) CAEP recordings and simulated signals.

#### Simulations

Data for the simulations consisted of simulated colored noise for representing the EEG background activity (generated as described in the specificity assessment above) and CAEP template waveforms for simulating a response. The CAEP templates were given by the coherently averaged waveforms where all three audiologists agreed that a clear CAEP was present, that is, a relatively strict criterion was used. This helps to ensure that the simulated CAEPs are indeed CAEPs, as opposed to background activity. A total of 45 CAEP template waveforms were available for the simulations, presented in Figure 1. For each ensemble of colored noise, a CAEP was simulated by randomly selecting one of the 45 templates, rescaling the template to obtain a certain SNR, and adding it to all epochs within the ensemble in question. The SNRs for the simulated CAEPs furthermore ranged from −20 to −8 dB, which covered the range of SNRs estimated from the infant CAEP recordings. The SNR was estimated using:

Eq. 9 $$\mathrm{SNR}=10\mathrm{lo}g_{10}\frac{p_{\mathrm{signal}}}{p_{\mathrm{noise}}}$$

where $p_{\mathrm{signal}}$ is the mean power of the coherently averaged epoch, and $p_{\mathrm{noise}}$ is the mean square of the unaveraged, continuous recording.

#### Infant CAEP Data

The audible CAEP recordings were divided into three categories, including the 0–10 dB SL, the 10.1–20 dB SL, and the >20 dB SL categories. A distinction was now also made between the MF and HF stimuli, but no distinction was made between test sessions. The recordings were again structured into ensembles of size *N*  =  20, 40, 80, or 160 epochs, and the 700 ms post-stimulus intervals were analyzed with the objective detection methods.

#### Post-hoc Statistical Analysis

Post-hoc analysis was carried out to test whether the detection rates of the methods differed when considered across all audible dB SL conditions, stimuli, and test sessions, but per ensemble size. For each detection method, the total number of detections (using α = 0.01) and non-detections were counted, after which Fisher's exact test (Fisher 1922; Fisher 1932) was used to test whether the number of detections and non-detections differed between methods, per ensemble size. The same approach was used when drawing comparisons between the detection methods and the examiners, except that there was now just a single ensemble size, equal to *N*  =  160.

### Test Reliability

This section evaluates intra- and inter-test reliability for the examiners and the objective detection methods. For CAEP detection, intra-test reliability—also known as test-retest reliability—is the extent to which a detection method or an examiner tends to reach the same conclusion in two separate recordings, where both recordings were obtained under the same test conditions, that is, the same test subject, the same stimulus type, stimulus rate, pre-processing parameters, etc. Inter-test reliability, on the other hand, is the extent to which detection methods and/or examiners agree on whether a CAEP is indeed present or not in any given recording. Both intra- and inter-test reliability were evaluated using Cohen's kappa statistic (Cohen, 1960; McHugh, 2012), further described below. In what follows, all “ambiguous” test outcomes for the examiners were treated as non-detections, giving a binary “CAEP detected” versus “no CAEP detected” test outcome. This facilitates the comparison between the examiners and the detection methods, as the detection methods similarly give a binary “H0 rejected” (CAEP detected) or “H0 not rejected” (no CAEP detected) test outcome.

#### Intra-Test Reliability

Data for the assessment comprised the infant CAEP recordings from sessions one and two (using *N*  =  160). Cohen's kappa (Cohen, 1960) was calculated, per detection method and per examiner, which represents the probability of reaching the same conclusion in both test sessions, after having adjusted for the probability of reaching the same conclusion by chance (McHugh, 2012). When calculating Cohen's kappa, it is helpful to first construct Table 2, which shows how often the test outcome from session 1 did, and did not, match with the test outcome from session 2 (see also the caption for Table 2). The observed probability of reaching the same conclusion in both sessions, say $P_{o}$, is then found using:

Eq. 10 $$P_{o}=\frac{C_{1,2}+C_{\neg 1\neg 2}}{n}$$

**Table 2.** Tables that were constructed when calculating Cohen's kappa statistic for the intra- and inter-test reliability assessment.

Intra-test reliability				Inter-test reliability			
		S1				M1	
		Yes	No			Yes	No
S2	Yes	$C_{1,2}$	$C_{\neg 1,2}$	M2	Yes	$C_{1,2}$	$C_{\neg 1,2}$
	No	$C_{1,\neg 2}$	$C_{\neg 1,\neg 2}$		No	$C_{1,\neg 2}$	$C_{\neg 1,\neg 2}$

For the intra-test assessment, each table indicates how often an ABR was detected in both session 1 (S1) and session 2 (S2) by some detection method or examiner, and is indicated by $C_{1,2}$. Similarly, $C_{\neg 1,\neg 2}$ indicates how often an ABR was not detected in both S1 and S2, $C_{1,\neg 2}$ is how often an ABR was detected in S1 but not in S2, and vice versa for $C_{\neg 1,2}$. For the inter-test assessment, the same notation was used, except that no distinction was made between test sessions. $C_{1,2}$ is now how often both Method 1 (M1) and Method 2 (M2) agreed that a CAEP was present, $C_{\neg 1,\neg 2}$ is how often both M1 and M2 agreed that a CAEP was not present, $C_{1,\neg 2}$ is how often M1 detected a CAEP when M2 did not, and vice versa for $C_{\neg 1,2}$.

where n is the total number of repeat recordings available for the analysis (here equal to 116), $C_{1,2}$ is the number of times an ABR was detected in both sessions 1 and 2, and $C_{\neg 1,\neg 2}$ is the number of times an ABR was not detected in both sessions 1 and 2. Next, the probability of reaching the same conclusion by chance, say $P_{c}$, is found using:

Eq. 11 $$P_{c}=\frac{A+B}{n}$$

where A and B are given by:

Eq. 12 $$A=\frac{\left(C_{1,1}+C_{1,\neg 2})\cdot (C_{1,1}+C_{\neg 1,2}\right)}{n}$$

Eq. 13 $$B=\frac{\left(C_{\neg 1,2}+C_{\neg 1\neg 2})\cdot (C_{1,\neg 2}+C_{\neg 1,\neg 2}\right)}{n}$$

and where $C_{1,\neg 2}$ is how often an ABR was detected in session 1 but not in session 2, and vice versa for $C_{\neg 1,2}$. Finally, Cohen's kappa is given by (Cohen, 1960; McHugh, 2012):

Eq. 14 $$\kappa =\frac{P_{o}-P_{c}}{1-P_{c}}$$

which takes values ranging from −1 to 1, with 1 representing perfect agreement, −1 perfect disagreement, and 0 the amount of agreement expected by chance.

#### Inter-Test Reliability

Data for the assessment again comprised the infant CAEP recordings, except that no distinction was made between test sessions. For the inter-test assessment, Cohen's kappa represents the extent to which detection methods and/or examiners agreed on whether a CAEP was present or not. It is again helpful to first construct Table 2 (right panel); Note that $C_{1,2}$ now refers to the number of ABRs that were detected by both methods and/or examiners, and similarly for $C_{\neg 1,\neg 2}$, $C_{1,\neg 2}$, and $C_{\neg 1,2}$ (see also Table 2 caption). For each pair of detection methods and/or examiners, Equations 10–14 were used to estimate the κ values. The total number of recordings available for the analysis (i.e., n) was now 232.

#### Post-hoc Statistical Analysis

To help interpret the estimated κ values, standard errors were constructed, per κ, using (McHugh, 2012):

Eq. 15 $$SE=\sqrt{\frac{P_{o}(1-P_{o})}{n{\left(1-P_{c}\right)}^{2}}}$$

giving 95% confidence intervals of κ ± 1.96·SE. For the intra-test reliability assessment, the 95% confidence intervals ranged from κ   ±  ∼0.16 to κ   ±  ∼0.21, whereas for the inter-test reliability assessment, the intervals ranged from κ   ±  ∼0.06 to κ   ±  ∼0.12.

## Results

This section presents the results from the specificity, sensitivity, and reliability assessments. Within the interest of remaining concise, results were not presented for all four q-sample modifications. Instead, just the best-performing modification was presented, which was QMod V3 (applied to phase values and amplitude ranks). The performance between all 4 q-sample modifications was, however, quite similar, and can be seen in Figure A.2. in the Supplemental Digital Content.

### Examiner Results

**Figure 2.**
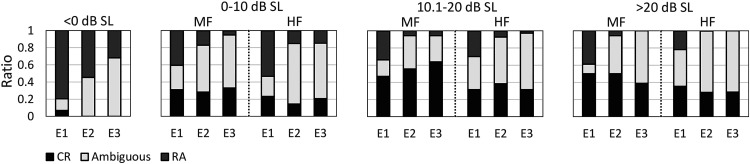
Results from the visual inspection by audiologists, presented as stacked-bar plots. Each bar represents the rates at which a clear response (CR) was detected, a response was deemed absent (response absent, or RA), and the waveform was deemed ambiguous in terms of CR or RA. Results are presented per dB SL category, for both the mid-frequency (MF) and the mid-high frequency (HF) stimulus, and for all three examiners, indicated by E1, E2, and E3. Note that for the <0 dB SL condition, no distinction was made between the MF and HF stimuli, as the stimuli were deemed inaudible.

The visual inspection results from the examiners are presented as stacked bar plots in Figure 2, which show the rates at which (1) a clear response (CR) was detected, (2) a response was deemed absent (response absent, or RA), or (3) the waveform was deemed ambiguous in terms of CR or RA. Results are presented for all three examiners (indicated by E1, E2, and E3), per dB SL category, and per stimulus type (i.e., MF or HF). Note that for the <0 dB SL category, no distinction was made between stimuli. If it is assumed that the inaudible recordings were indeed inaudible and did not contain a CAEP, then results show true-negative rates (TNRs; it is correctly concluded that a CAEP was absent) ranging from ∼0.3 (for E3) to ∼0.8 (for E1). For the audible test conditions, non-detection rates ranged from 0 to ∼0.55. Whether these non-detections can be considered as false-negative (i.e., it is concluded that a CAEP was absent when a CAEP was in fact present) is not clear—see also the Discussion. Detection rates for the inaudible recordings (assumed to be FPRs) were 0.068 for E1 and 0 for both E2 and E3. For the audible recordings, detection rates ranged from ∼0.15 to ∼0.65.

### Test Specificity

*Simulations.* The FPRs of the detection methods (Table 3) mostly fell within the 99% confidence intervals for α = 0.01. The exception was QMod V3, which showed a liberal (FPR > α = 0.01) test performance at *N*  =  40. In general, FPRs show a small bias towards a liberal test performance for the bootstrapped test statistics at small ensemble sizes of *N*  =  20 and *N*  =  40, which decreases for increasing *N,* and can likely be attributed to variance in the power spectral density estimates ($\hat{P}j$) used in the frequency domain bootstrap. To ensure a fair comparison in the subsequent assessments of test sensitivity, the nominal α-levels were adjusted, per test statistic, such that their FPRs equal 0.01. The adjusted α-levels are also shown in Table 3 (middle panel).

**Table 3.** False-positive rates (FPRs) for the objective detection methods in simulations (left panel) and for the inaudible (<0 dB SL) infant CAEP recordings (right panel).

	FPRs simulations				Adjusted α-levels simulations				FPRs infant data			
	*N* = 20	*N* = 40	*N* = 80	*N* = 160	*N* = 20	*N* = 40	*N* = 80	*N* = 160	*N* = 20	*N* = 40	*N* = 80	*N* = 160
**T^2^_Time_**	0.01	0.0107	0.0081	0.0089	0.01	0.01	0.012	0.012	0.006	0	0	0
**T^2^_Freq_**	0.0089	0.0105	0.0081	0.0089	0.01	0.01	0.012	0.012	0.023	0	0	0
**QMod V3 (b)**	0.0122	0.0129*	0.0109	0.0116	0.008	0.008	0.008	0.008	0.009	0.006	0	0
**T^2^_Toep_ (b)**	0.0112	0.0124	0.0088	0.0115	0.008	0.008	0.012	0.008	0.006	0.006	0.012	0
**T^2^_Diag_ (b)**	0.0126	0.0124	0.0104	0.0109	0.008	0.008	0.01	0.008	0.02	0	0	0.023

Significant (*p* < .01) deviations from α = 0.01 are indicated by an asterisk to ensure that the comparison in test sensitivity in subsequent analyses were fair, the nominal α-levels were adjusted, per detection method, such that their FPRs equal 0.01 (middle panel). The (b) indicates that test significance was evaluated using the frequency domain bootstrap.

*Infant data*. The FPRs of the detection methods for the inaudible infant CAEP recordings are also shown in Table 3 (right panel). All FPRs fell within the expected 99% confidence intervals for α  =  0.01. However, it is worth emphasizing that there was relatively little data for the assessment, which resulted in wide confidence intervals for α  =  0.01. This may have prevented small deviations from the α-levels from being detected.

### Test Sensitivity

**Figure 3.**
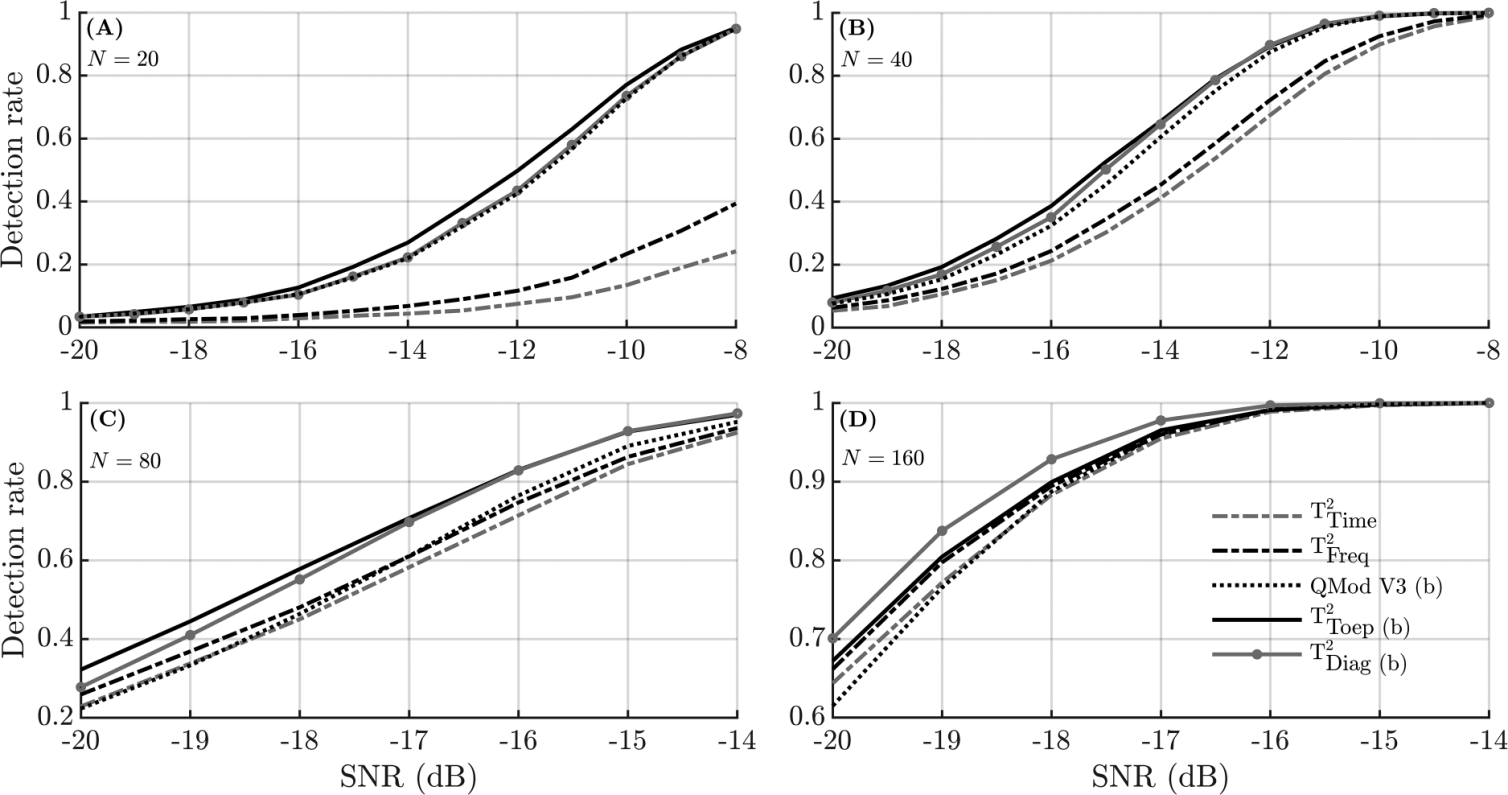
Simulation results showing the detection rates as a function of the SNR for different ensemble sizes *N*. To ensure a fair comparison between methods, the detection rates were generated using adjusted α-levels (Table 3). Note that the *y*-axis and *x*-axis for panels (C) and (D) are on a different scale than panels (A) and (B). The (b) in the legend indicates that test significance was evaluated using the frequency domain bootstrap.

*Simulations.* The detection rates using the adjusted α-levels are shown in Figure 3 for *N*  =  20, 40, 80, and 160. Results show that detection rates for the conventional HT^2^ test were relatively poor for *N*  =  20 and *N*  =  40 (panels A and B, respectively), but improved for larger ensemble sizes: For *N*  =  80, similar detection rates were observed for HT^2^ and QMod V3, whereas for *N*  =  160, HT^2^ outperformed QMod V3. The highest detection rates were consistently observed for the modified T^2^ statistics.

**Figure 4.**
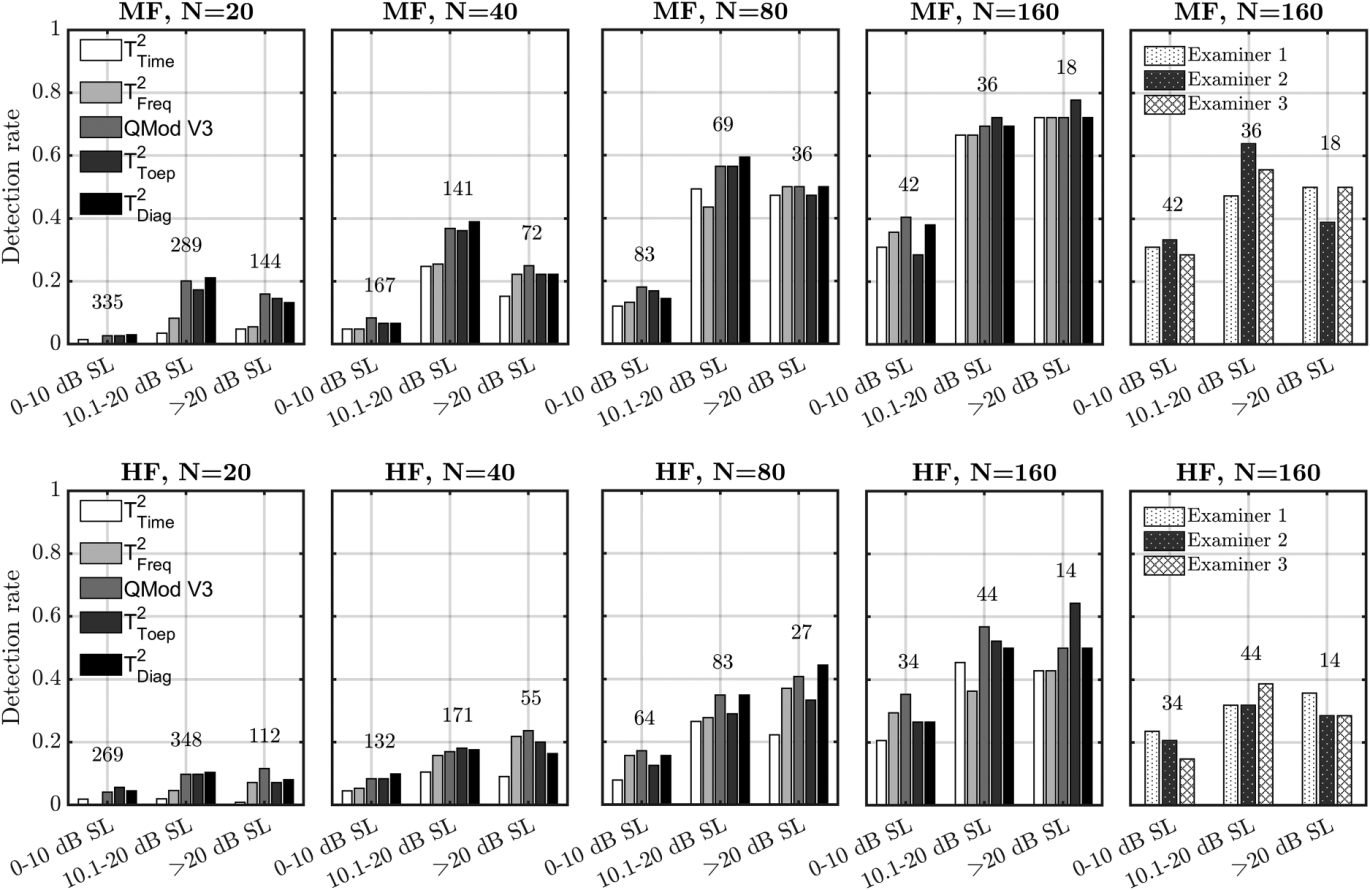
Detection rates for the audible infant CAEP recordings for both the objective detection methods and the examiners. Results are presented for the mid-frequency (MF) stimuli (top panels) and the mid-high frequency (HF) stimuli (bottom panels), per dB SL category. The numbers associated with each dB SL category indicate how many ensembles were tested.

*Infant data.* The detection rates for the objective detection methods are presented per dB SL category and per ensemble size in Figure 4, for both the MF stimulus (top panels) and the HF stimulus (bottom panels). For the smaller ensembles sizes of *N*  =  20 and *N*  =  40, results again suggest that the modified T^2^ statistics and QMod V3 outperformed the conventional HT^2^ test (i.e., T^2^_Time_ and T^2^_Freq_), which was confirmed by results from the post-hoc statistical analysis, shown in Table 4. For *N*  =  80, T^2^_time_ was also significantly (*p* < .05) outperformed by both QMod V3 and T^2^_Diag_, whereas for *N*  =  160, no significant differences between the detection rates were observed (*p* > .05).

**Table 4.** Results from the post-hoc statistical analysis for the sensitivity assessment, i.e., the *p*-values generated by Fisher's exact test (section “Test Sensitivity”) when comparing the detection rates amongst objective detection, and between detection methods and examiners.

	T^2^_Time_	T^2^_Freq_	QMod V3 (b)	T^2^_Toep_ (b)	T^2^_Diag_ (b)
	***N* = 20**				
**T^2^_Time_**	1	0.008*	<0.001*	<0.001*	<0.001*
**T^2^_Freq_**		1	<0.001*	<0.001*	<0.001*
**QMod V3 (b)**			1	0.5	0.9
**T^2^_Toep_ (b)**				1	0.39
**T^2^_Diag_ (b)**					1
	***N* = 40**				
**T^2^_Time_**	1	0.091	<0.001*	0.005*	0.003*
**T^2^_Freq_**		1	0.033*	0.082	0.061
**QMod V3 (b)**			1	0.742	0.844
**T^2^_Toep_ (b)**				1	0.95
**T^2^_Diag_ (b)**					1
	***N* = 80**				
**T^2^_Time_**	1	0.571	0.029*	0.177	0.035*
**T^2^_Freq_**		1	0.123	0.48	0.142
**QMod V3 (b)**			1	0.45	1
**T^2^_Toep_ (b)**				1	0.493
**T^2^_Diag_ (b)**					1
	***N* *=* *160***				
**T^2^_Time_**	1	1	0.154	0.389	0.389
**T^2^_Freq_**		1	0.184	0.45	0.45
**QMod V3 (b)**			1	0.64	0.64
**T^2^_Toep_ (b)**				1	1
**T^2^_Diag_ (b)**					1
**Examiner 1**	0.112	0.091	0.002*	0.011*	0.011*
**Examiner 2**	0.198	0.167	0.005*	0.025*	0.025*
**Examiner 3**	0.136	0.112	0.003*	0.015*	0.015*

This analysis considered all audible (≥0 dB SL) CAEP recordings simultaneously and made no distinction between sessions or stimulus type. All pair-wise comparisons with significantly (*p* < .05) different detection rates are indicated by an asterisk. Results show that T^2^_Time_ and T^2^_Freq_ were significantly outperformed by T^2^_Toep,_ T^2^_Diag_, and QMod V3, primarily at *N*  =  20 and 40. The (b) indicates that test significance was evaluated using the frequency domain bootstrap.

To help facilitate the comparison between the examiners and the detection methods (at *N*  =  160), examiner detection rates from Figure 2 were also included in Figure 4 (right panels). Examiner detection rates ranged from ∼0.15 to ∼0.4 for the HF stimulus, and from ∼0.3 to ∼0.65 for the MF stimulus. For the best-performing detection methods (i.e., the modified T^2^ statistics and QMod V3), detection rates for *N*  =  160 were in the ∼0.2 to ∼0.65 range for the HF stimuli, and in the ∼0.3 to ∼0.8 range for the MF stimuli. Results from the post-hoc statistical analysis (Table 4) confirm significantly higher detection rates for QMod V3, T^2^_Diag_, and T^2^_Toep_ relative to the examiners.

Finally, increasing the α-level to 0.05 for the objective detection methods resulted in detection rates ranging from ∼0.45 to ∼0.75 for the HF stimuli, and from ∼0.45 to ∼0.9 for the MF stimuli. Additional analysis (details not presented) also demonstrate similar detection rates when using both the adjusted and the non-adjusted α-levels. Results in Figure 4 were generated using the non-adjusted α-levels.

### Test Reliability

Results from the reliability assessment are presented in Table 5, and show Cohen's kappa values for the intra- and inter-test reliability assessments. For the intra-test reliability assessment (i.e., test-retest reliability), κ values ranged from just 0.16 for Examiner 1, up to 0.49 for T^2^_Time_. This suggests that the test outcome from session one was a relatively poor predictor for the test outcome for session two. The 95% confidence intervals ranged from κ   ±  ∼0.16 to κ   ±  ∼0.21, and suggest a significantly (*p* < .05) lower intra-test reliability for Examiner 1 relative to T^2^_Time_, T^2^_Freq_, T^2^_Diag_, and T^2^_Toep._ For the inter-test reliability assessment, κ values ranged from 0.48 to 0.9. Results also suggest that agreement amongst examiners was generally lower than agreement amongst detection methods, that is, κ values for the examiners ranged from 0.65 to 0.71, whereas for the detection methods κ values ranged from 0.72 to 0.9. The lowest κ values, however, were observed when considering the amount of agreement between detection methods and examiners: κ values now ranged from 0.48 to 0.64 (see also the Discussion). For all κ values, the 99% confidence intervals ranged from κ   ±  ∼0.06 to κ   ±  ∼0.12, thus indicating many significant (*p* < .05) differences in the level of agreement amongst detection methods and examiners.

**Table 5.** Cohen's Kappa values from the intra- and inter-test reliability assessments.

	*Intra-test reliability*							
	**T^2^_Time_**	**T^2^_Freq_**	**QMod V3**	**T^2^_Toep_**	**T^2^_Diag_**	**E1**	**E2**	**E3**
	0.49	0.48	0.35	0.48	0.41	0.16	0.35	0.32
	*Inter-test reliability*							
	**T^2^_Time_**	**T^2^_Freq_**	**QMod V3**	**T^2^_Toep_**	**T^2^_Diag_**	**E1**	**E2**	**E3**
**T^2^_Time_**	1	0.90	0.80	0.80	0.85	0.55	0.63	0.61
**T^2^_Freq_**		1	0.79	0.77	0.83	0.54	0.62	0.62
**QMod V3**			1	0.72	0.89	0.54	0.58	0.63
**T^2^_Toep_**				1	0.77	0.48	0.57	0.57
**T^2^_Diag_**					1	0.59	0.64	0.64
**E1**						1	0.65	0.67
**E2**							1	0.71
**E3**								1

For the intra-test reliability assessment, κ indicates how consistent a detection method or examiner is at arriving at the same conclusion when presented with a repeat recording. For the inter-test reliability assessment, κ represents the amount of agreement amongst examiners and/or detection methods when determining whether a CAEP is present or not. Results generally show higher κ values for the detection methods relative to the examiners—further considered in the main text.

## Discussion

This study evaluated various new and existing objective methods for aided CAEP detection in infants with hearing loss, and aimed to improve the accuracy and efficiency of CAEP measurements in this population. Results firstly confirm the “large p small n” problem (Li et al., 2017) for the HT^2^ test, which was previously hypothesized to be due to poor estimates of the feature covariance matrix (Bai & Saranadasa, 1996). Indeed, improved test sensitivities were obtained by replacing the conventional feature covariance matrix with either the Toeplitz (Equation 4) or the diagonal (Equation 5) covariance matrix.

Some alternative modifications for preventing low HT^2^ test sensitivities have previously also been proposed in the literature. These have been categorized by Dong et al. (2016) as: (1) the “unscaled T^2^ statistics” where the feature covariance matrix is removed from the T^2^ statistic (e.g., Bai & Saranadasa, 1996; Zhang & Xu, 2009), (2) the “regularized T^2^ statistics” where a regularization factor is applied to the feature covariance matrix (Chen et al., 2011), and (3) the “diagonal T^2^ statistics” where all covariances are set to zero, giving a diagonal covariance matrix (e.g., Witten & Tibshirani, 2011). The T^2^_Diag_ statistic proposed in the current work falls in the third category, but is specifically justified for the frequency domain HT^2^ test due to statistics of the Fourier Transform coefficients from stationary signals. The T^2^_Toep_ statistic falls outside this classification scheme, but is again based on prior knowledge (or the assumption) of signal stationarity.

### Alternative Detection Methods

Other detection methods published in the literature were initially also included in the assessment, but were removed from the final results to remain concise. These include various alternative modified q-sample statistics from Cebulla et al. (2006), the Fsp (Elberling & Don, 1984), the Fmp (Martin et al., 1994), the “mean power” statistic (Lv et al., 2007), the DTW approach (Chesnaye et al., 2021a), and the diagonal HT^2^ test from Bai and Saranadasa (1996).

Starting with the diagonal HT^2^ test (Bai & Saranadasa, 1996), this approach—not previously evaluated for CAEP detection—is essentially the conventional time domain HT^2^ test where the feature covariance matrix is replaced with the diagonal covariance matrix, that is, covariances between all voltage means are set to zero. The approach also includes a rescaling factor for transforming the modified T^2^ statistic into a standard normally-distributed random variable, after which the asymptotic (for N,Q→∞) null distribution is given by the standard distribution (theorem 2.1 in Bai & Saranadasa, 1996). Results from additional simulations (details not presented) suggest that the standard distribution is indeed a close approximation, but still slightly inaccurate when using typical values for *Q* and *N*. Using *Q*  =  14 and *N*  =  50, for example, resulted in a slightly liberal FPR of ∼0.014 for α=0.01. Test sensitivity for the diagonal HT^2^ test from Bai & Saranadasa (1996) was also slightly lower than the modified T^2^ and q-sample statistics from the current study, and was therefore excluded from the final results. However, it is worth noting that the diagonal HT^2^ test from Bai & Saranadasa (1996) does not require a bootstrap to evaluate test significance, and is therefore computationally less demanding than the modified test statistics from the current study for which the bootstrap (or similar surrogate data method) is required.

In a previous study on CAEP detection in adults with normal hearing, the best-performing method was a DTW approach, which correlates the (time-warped) ensemble coherent average to an a priori assumed CAEP template (Chesnaye et al., 2021a). Due to the wide variation in CAEP waveforms across infants (Figure 1), the DTW approach was not expected to perform optimally in this young population. Indeed, detection rates for the DTW approach were around 0.4 or lower in the infant data, even at *N*  =  160, and detailed results were excluded from the results section. We also attempted a range of template matching variants, such as using a database of templates, but none provided encouraging results.

### Test Specificity, Sensitivity, and Reliability

Results from visual inspection by examiners show good test specificities but relatively low test sensitivities, that is, detection rates (using *N*  =  160) for the audible (≥0 dB SL) CAEP recordings were in the ∼0.3 to ∼0.65 range for the MF stimuli, and in the ∼0.15 to ∼0.4 range for the HF stimuli. The low test sensitivity for visual inspection appears potentially problematic for clinical decision making with aided CAEP. For the best-performing objective detection methods, detection rates for *N*  =  160 were slightly higher, for example, T^2^_Toep_ had detection rates in the ∼0.25 to ∼0.65 range for the HF stimulus, and in the ∼0.3 to ∼0.8 range for the MF stimulus. Increasing the α-level to 0.01 from 0.05 further resulted in slightly higher detection rates, at the cost of a reduced test specificity.

Detection rates in this study were approximately in the same range as those observed in previous studies. For example, Van Dun et al. (2012) observed detection rates (using the conventional HT^2^ test) for aided CAEPs in hearing-impaired infants of ∼0.53 for the 0–9.9 dB SL category, ∼0.67 for the 10–19.9 dB category, and ∼0.77 for the 20  +  dB SL category. Similarly, Chang et al. (2012) observed detection rates (using the conventional HT^2^ test) of ∼0.63 for the >0 dB SL category, ∼0.68 for the >10 dB SL category, and ∼0.69 for the >20 dB SL category, for aided and unaided CAEP detection in hearing-impaired infants. Note that these studies used an α-level of 0.05, whereas the current study used an α-level of 0.01.

With respect to intra-test reliability (or test re-test reliability), relatively low κ values were observed for both the objective detection methods and the examiners (κ values ranged from 0.16 to 0.49). This indicates that the test outcome (i.e., CAEP present or not) often changed from one session to the next, which, in turn, suggests that CAEPs might not always have been successfully evoked in all recordings. The latter is supported by results from Visram et al. (under review), who carried out a more in-depth assessment of test re-test reliability, and note that, “in 42% of the cases where the stimulus was audible, but the CAEP was not detected at the initial test, the CAEP *was* detected on retest.” Visram et al. accordingly emphasize that non-detections should be interpreted with care, even when test conditions appear good. These results underline the challenge in efficiently and reliably evoking CAEPs in infants with hearing loss.

For the inter-test reliability assessment, κ values were considerably higher. Note again that κ now represents the level of agreement (in terms of CAEP present or not) between pairs of detection methods and/or examiners. The highest κ values were observed when comparing the detection methods (κ ranged from 0.72 to 0.9), which can be attributed to the features (i.e., the actual data analyzed by the detection methods) being highly correlated. For example, the T^2^_Time_ and T^2^_Toep_ detection methods both consider the same voltage means, and only differ in terms of the feature covariance matrix. Similarly, T^2^_Freq_, and T^2^_Diag_ both consider the real and imaginary parts of the same spectral bands, and differ only in terms of the feature covariance matrix. Finally, QMod V3 excludes the feature covariance matrix altogether, and is applied to the phases and magnitude (ranks) of the spectral bands, as opposed to the real and imaginary parts, and thus also has considerable overlap with T^2^_Freq_, and T^2^_Diag_.

Finally, inter-test reliability scores for the examiners were lower than those of the objective detection methods (κ ranged from 0.65 to 0.71). This suggests that examiners varied, to some extent, in how they chose to evaluate the waveforms. The lowest reliability scores, however, were observed when pairing examiners with detection methods (κ ranged from 0.48 to 0.64), which suggests that examiners and detection methods differed in terms of what information they considered when evaluating CAEPs. One hypothesis is that examiners strove to identify credible morphologies in the coherently averaged waveforms, whereas the objective detection methods did not. In some cases, this may have led to CAEPs with small amplitudes, but credible waveform morphologies, being detected by the examiners, but being classified as non-detections by the detection methods. Vice versa, some CAEPs with less conventional morphologies may have been detected by the detection methods, but classified as non-detections by the examiners.

### Study Limitations and Future Work

This study aimed to provide a fair comparison between aided CAEP detection methods, and therefore carried out feature optimizations (presented in the Supplemental Digital Content) prior to the main assessments. Feature optimizations prevent some methods from having an unfair advantage over others due to sub-optimal feature sets, but have a potential risk in that some methods have more capacity to be optimized than others, that is, some methods may have been overfitted to the sample of CAEP recordings. Although results in the Appendix suggest that test performance was relatively robust across a range of test parameters, it is important that results are confirmed using alternative data sets in future work.

An additional limitation for this work is that there were relatively few inaudible (<0 dB SL) CAEP recordings available for the specificity assessment. As a result, statistical power was low, which may have resulted in small biases in the FPRs of the detection methods going undetected. The large sample of simulated signals overcomes this limitation to some extent, but does not fully emulate real-world recordings, and was not carried out for visual inspection due to the substantial load on the clinicians. In future work, it may be necessary to estimate FPRs for the detection methods and examiners using a much larger sample of no-stimulus recordings.

It should also be noted that any longitudinal studies that compare tests on children at different ages (in this case CAEP testing at 3–7 months followed by VRA testing at mean age 10.8 months), and where the tests have some source of variability, have inherent limitations. Both VRA and CAEP have some test variability due to the attention of the child and measurement noise resulting from child movement, that is, neither test is entirely a gold standard measurement of audibility. Although the current study strived to reduce sources of variability in measurements—e.g., by (1) carrying out otoscopy and tympanometry examinations to rule out variations due to conductive elements, (2) excluding infants with progressive or fluctuating hearing loss, and (3) testing hearing aids using a test box prior to the examinations—data should be treated with some caution.

Finally, the detection methods in this study made no distinction between epochs within an ensemble, and therefore implicitly assumed that the CAEP was a deterministic response, unchanging throughout the examination. However, the CAEP is known to be affected by habituation (Özesmio et al., 2000) and subjects’ drowsiness or state of alertness (e.g., Celesia & Puletti, 1971; Johnson & Yonovitz, 2007), thus rendering significant trial-to-trial variations plausible. Indeed, Johnson & Yonovitz (2007) observed gradual changes in CAEP amplitudes and latencies over the course of a 90-min test paradigm and found these changes to be associated with fluctuations in alertness and attentiveness. Further work is required to explore these variations in infants with hearing loss, quantify their impact on CAEP detection methods, and test potential solutions.

## Conclusion

The overall best-performing methods to detect CAEPs in this study were the modified T^2^ statistics, which outperformed QMod V3 in simulations, and the conventional HT^2^ test in both simulations and aided CAEP recordings from 59 hearing-impaired infants. The reduced test sensitivity for the conventional HT^2^ test was primarily for small ensemble sizes, and was attributed to the “small n large p” problem underlying HT^2^. For larger ensemble sizes of 80 epochs or more, the low test sensitivity was less pronounced. With respect to the visual inspection results of the examiners, good test specificities were observed, but relatively low test sensitivities, which could be problematic for clinical applications. Finally, results from the reliability assessment suggest that some audible CAEP recordings might not have contained clear CAEP waveforms. Future developments in CAEP detection might therefore aim to optimize CAEP test paradigms for efficiently and reliably evoking CAEPs in infants with hearing loss, and to further improve objective CAEP detection methods to assist clinicians with interpreting the CAEP waveforms.

## Supplemental Material

sj-docx-1-tia-10.1177_23312165231154035 - Supplemental material for Modified T^2^ Statistics for Improved Detection of Aided Cortical Auditory Evoked Potentials in Hearing-Impaired Infants Click here for additional data file. Supplemental material, sj-docx-1-tia-10.1177_23312165231154035 for Modified T^2^ Statistics for Improved Detection of Aided Cortical Auditory Evoked Potentials in Hearing-Impaired Infants by Michael Alexander Chesnaye, Steven Lewis Bell, James Michael Harte, Lisbeth Birkelund Simonsen, Anisa Sadru Visram, Michael Anthony Stone, Kevin James Munro and David Martin Simpson in Trends in Hearing
