# Supplementary material for: Modified T2 Statistics for Improved Detection of Aided Cortical Auditory Evoked Potentials in Hearing-Impaired Infants
Source: Trends Hear. 2023 Feb 27;27:23312165231154035. doi: 10.1177/23312165231154035 (PMC9974628; doi:10.1177/23312165231154035)
Supplement: sj-docx-1-tia-10.1177_23312165231154035 - Supplemental material for Modified T2 Statistics for Improved Detection of Aided Cortical Auditory Evoked Potentials in Hearing-Impaired Infants [file sj-docx-1-tia-10.1177_23312165231154035.docx]

**Appendix**

To prevent an unfair comparison between detection methods due to sub-optimal feature sets, feature optimisations were carried out using simulations prior to the analyses documented in the main paper. The simulated data consisted of coloured noise to represent the EEG background activity and CAEP templates for simulating response, as described in the main text (Section 2). A total of 5,000 ensembles were simulated for both the stimulus and no-stimulus condition. The ensemble size was set to *N*=60 epochs, and the SNR for the simulated CAEP was set to -16.2 dB, which was the mean estimated SNR of the infant CAEP waveforms where a response was deemed present by all three audiologists.

Test performance was evaluated using Received Operator Characteristic (ROC) curves (Fawcett, 2006), which show the true-positive rate (TPR) as a function of the false-positive rate (FPR) for α-levels ranging from 0 to 1. When interpreting the ROC curve, it is useful to define two extremes. At one extreme, the ROC curve follows the diagonal line, from [0,0] to [1,1], which implies that the TPRs and FPRs are equivalent for all α-levels, i.e. classification is equivalent to purely random allocation. At the other extreme, the ROC curve follows a 90° angle, from [0,0], to [0,1], to [1,1]. In this case, the test is the ideal test, and can always discriminate perfectly between the stimulus and no-stimulus recordings.

A useful measure for evaluating the ROC is the Area Under the Curve, or AUC. The AUC is defined quite literally as the area under the ROC curve, and represents a single measure of the test’s ability to distinguish between the stimulus and no-stimulus recordings across all α-levels (Fawcett, 2006). An AUC of 1 represents perfect discriminatory capacity (the ideal test), whereas an AUC of 0.5 indicates no discriminatory capacity, or random test performance. The AUC was approximated in this work through numerical integration of the ROC curve.

The remainder of this document describes the optimisation procedures for the detection methods, which include the conventional Hotelling’s T^2^ (HT2) test applied in the time and frequency domains, the modified q-sample statistics, and the modified T^2^ statistics.

**The time domain Hotelling’s T^2^ test**

For the time domain Hotelling’s T^2^ test, or T^2^_Time_, the aim was to optimise the number of voltage means (i.e. Q) to extract from the 0-700 ms post stimulus intervals. The optimal choice for *Q* depends on several factors. Firstly, when *Q* is small, then voltage means are taken across relatively large time intervals, potentially resulting in consecutive peaks and troughs in the CAEP waveforms cancelling out, and a loss of information. As *Q* is increased, more information is retained, which comes at the cost of consecutive voltage means becoming increasingly correlated, along with an increasingly ill-conditioned covariance matrix $\text{S}$. The latter may result in $\text{S}^{-1}$ being contaminated with calculation errors, or in the extreme case (for *Q* ≥ *N*), in $\text{S}^{-1}$ being singular. The third factor to consider is the ensemble size, which is again related to the “small n large p” problem and its adverse effect on test sensitivity due to poor covariance matrix estimates (Bai & Saranadasa, 1996). In what follows, test performance was therefore evaluated for different Q, which was varied from 1 to 30. Test significance was evaluated using either conventional F-distributions or the frequency domain bootstrap (FDB).

Results

The AUCs are presented as a function of the number of voltage means Q in Figure A.1 below. Results firstly demonstrate a similar test performance between T^2^_Time_ evaluated with F-distributions and T^2^_Time_ evaluated using the FDB, which confirms that test performance was not significantly impacted by the FDB, albeit for this particular data set. The largest AUCs were observed when using approximately 14 voltage means, which corresponds to averaging across 50 ms time intervals. For the analyses in the main text, *Q*=14 voltage means were therefore used.


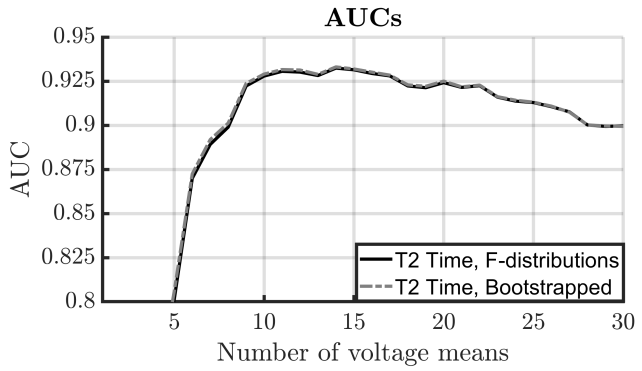


**Figure A.1.** The Area Under the Receiver Operator Characteristic Curve (AUC) as a function of the number of voltage means for the conventional T^2^ statistic where test significance was evaluated using either theoretical F-distributions or bootstrapped distributions generated by the frequency domain bootstrap.

**The frequency domain Hotelling’s T^2^ test and the modified Q-sample statistics**

For the frequency domain Hotelling’s T^2^ test (T^2^_Freq_) and the modified Q-sample (QMod) statistics, the aim was to determine which spectral bands to include in the analysis. All spectral bands between 1 and 15 Hz were first ranked according to a mean t statistic, defined as follows:

where K is the number of ensembles (all with *N*=160 epochs) that contain a clear response (45 total), and are the means of the real and imaginary parts, respectively, calculated from the f^th^ spectral band of the k^th^ ensemble, and $\sigma_{\mathrm{Rkf}}$ and $\sigma_{\mathrm{Ikf}}$ are the standard deviations of the real and imaginary parts, respectively, again calculated from the f^th^ spectral band of the k^th^ ensemble. This resulted in the following ranking of spectral bands: 5.7, 4.3, 7.1, 2.9, 1.4, 8.6, 10, 12.9, 11.4, and 14.3 Hz (see also Figure A.2 below). After ranking, simulations were carried out using the top *W*-ranking spectral bands, where *W* was varied from 1 to 10. For example, when using *W*=2, the frequency domain detection methods were applied to the 5.7 and the 4.3 Hz bands, whereas when using *W*=3, they were applied to the 5.7, 4.3, 7.1 Hz bands, etc.

Results

The mean t statistic is first plotted as a function of the frequency bands in Figure A.2 (left panel). The AUCs of the detection methods are then plotted as a function of the number of top-ranking spectral bands *W* that were included in the analysis, also in Figure A.2 (right panel). Note again that QMod V1 is applied to phase ranks and magnitude ranks, QMod V2 to phase ranks and magnitude values, QMod V3 to phase values and magnitude ranks, and QMod V4 to phase values and magnitude values (Cebulla et al, 2006; see also Section 2 in the main text). For the QMod statistics, test performance flattened out after *W*=7, whereas for T^2^_Freq_, test performance started to decrease after *W*=6. For the main analyses, the QMod statistics were therefore applied to the 7 top-ranking spectral bands, whereas T^2^_Freq_ was applied to the 6 top-ranking spectral bands.


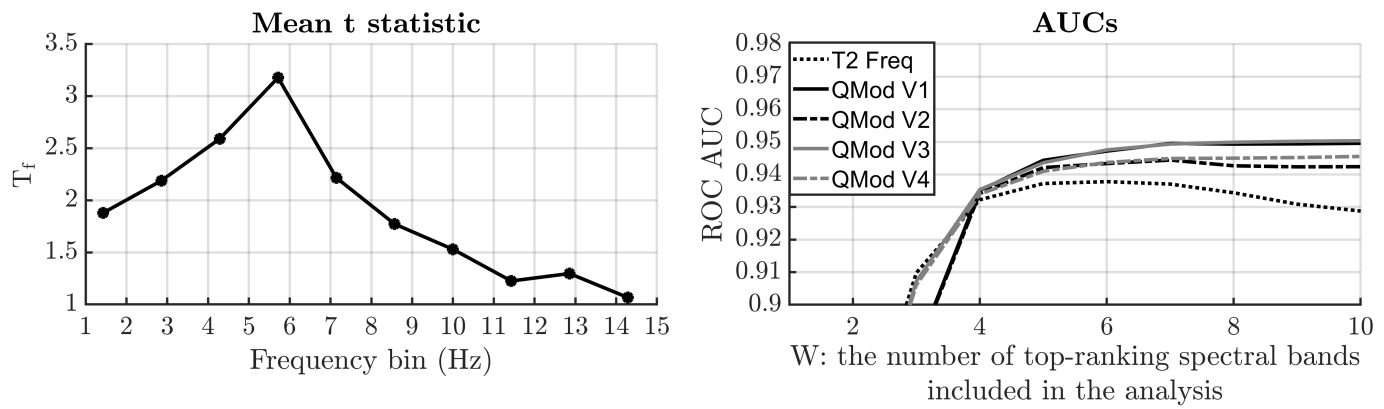


**Figure A.2.** The mean t statistic as a function of the frequency bin (left panel) and the AUCs of the detection methods, as a function of the number of top-ranking spectral bands *W* that were included in the analysis (right panel). The lower test sensitivity for T2_Freq_ relative to the QMod statistics can likely be attributed to the “small n large p” problem underlying the HT2 test, as discussed in the main document.

**Modified T^2^ statistics: T^2^_Toep_**

For the T^2^_toep_ statistic, the number of voltage means *Q* to extract from the 0-700 ms post stimulus intervals was varied from 1 to 50, but in irregularly increasing steps, i.e. an additional requirement for *Q* was that the number of samples within the analysis window was integer-divisible by *Q*. This was to ensure that the time-windows across which the voltage means were calculated all had the same duration, as is assumed by the autocovariance function (*Eq. 2* in main text) from which the feature covariance matrix for T^2^_Toep_ was derived.

Results

The AUCs are plotted as a function of the number of voltage means *Q* in Figure A.3 below. Note that, as *Q* is increased, features become more correlated, resulting in an increasingly ill-conditioned feature covariance matrix. The latter can, in some cases, introduce calculation errors when taking the inverse of the feature covariance matrix, which accounts for the increasingly erratic test performance for *Q*>25. For the main analyses, it was opted to stay well within the range where test performance was stable, and to use *Q*=14 voltage means.

**
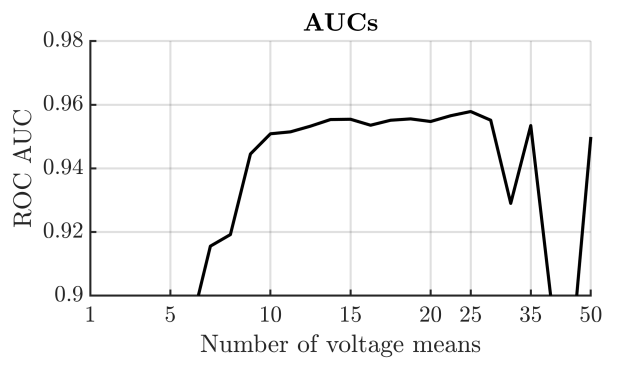
**

**Figure A.3.** The ROC AUCs for T^2^_Toep_ as a function of the number of voltage means *Q*. Note that *Q* was varied in irregular steps, as described in the text above.

**Modified T2 statistics: T^2^_Diag_**

For the T^2^_Diag_ statistic, the aim was to optimise the weight vector **w** (see *Eq. 7* in main text) for the 1.4, 2.9, 4.3, 5.7, 7.1, 8.6, 10, 11.4, 12.9 and 14.3 Hz bands. Note that each frequency band received a single weight, which was applied to both the real and imaginary parts of the Fourier components of that frequency band. To reduce processing time, weights were optimised sequentially across frequency bands. To do so, frequency bands were first ranked according to their mean estimated estimated t statistic (directly related to SNR), as described above. The weight for the first band (5.7 Hz) was then set to one. The second top-ranking frequency band was then also included in the analysis (the 4.3 Hz band), and its weight was varied from 0 to 1.5, in steps of 0.01. For each weight, an AUC was calculated, after which the weight with the largest AUC was assigned to the second top-ranking spectral band. This procedure was repeated for until the weights for all 10 spectral bands had been optimised.

Results

The AUCs are plotted per frequency band as a function of their weighting in Figure A.4 below. The optimal weight for each frequency band is indicated by a grey dot. The optimal weights are given per spectral band in Table A.1. below. In an attempt to prevent overfitting, the following general rules of thumb were applied: (1) all frequency bands between 1 and 8 Hz received a weight of 1, (2) all bands between 8 and 9 Hz received a weight of 0.75, and (3) all bands between 9 and 15 Hz received a weight of 0.5.


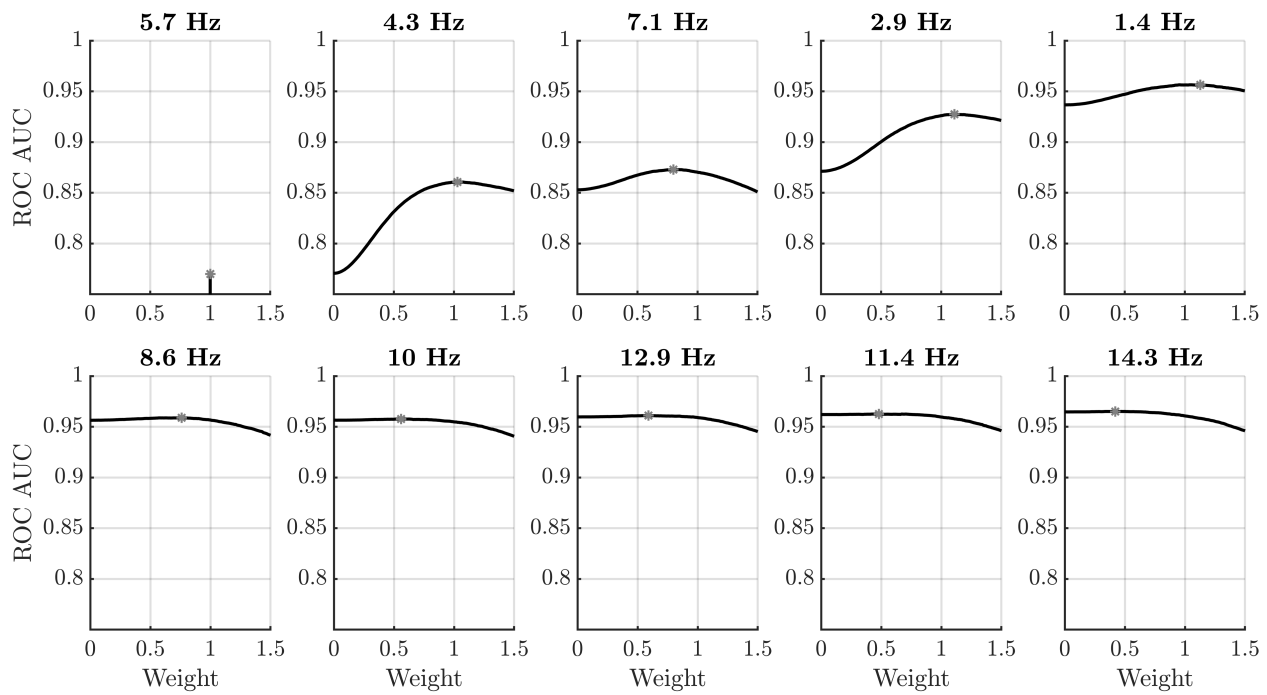


**Figure A.4.** The AUCs per frequency band as a function of the frequency band-specific weighting. The grey dots indicate the weights associated with the highest AUCs.

| **Band (Hz)** | 5.7 | 4.3 | 7.1 | 2.9 | 1.4 | 8.6 | 10 | 12.9 | 11.4 | 14.3 |
| --- | --- | --- | --- | --- | --- | --- | --- | --- | --- | --- |
| **Weights** | 1 | 1.03 | 0.8 | 1.11 | 1.13 | 0.76 | 0.56 | 0.59 | 0.48 | 0.42 |

**Table A.1.** The optimised weights, per spectral band, for the T^2^_Daig_ statistic.

Discussion

Results show that test sensitivity was impacted primarily by the 5 top-ranking spectral bands, which includes all bands between 1 and 8 Hz. Test performance was furthermore optimal when these bands received a more or less equal weighting. Including the 8-15 Hz bands had relatively little impact on test performance, albeit under the condition that these were down-weighted using weights of ~0.5 or less. These results hence suggest that, when applied to all spectral bands between 1 and 8 Hz, the weight vector **w** can potentially be removed from the T^2^_Diag_ statistic, and all spectral bands can be weighted equally. Within the interest of a fair comparison between methods, the analysis in the main text used the optimised weight vector **w*.***

**References**

Bai Z., Saranadasa H. Effect of high dimension: by an example of a two-sample problem. Statistica Sinica, (1996). 6(2), 311-329.

Fawcett T. 2006. An introduction to ROC analysis. Pattern Recognition Letters, Vol 27, pp. 861-874. DOI: 10.1016/j.patrec.2005.10.010
